# Controlled Intramedullary Pressure Fluctuations Enhanced Periosteal and Endosteal Bone Formation in Young, but Not Old, Fischer-344 Rats

**DOI:** 10.3390/biomimetics11070504

**Published:** 2026-07-18

**Authors:** Najmeh Sadat Hosseini, Amanda Salas Sanchez, Teresa Le, Sunggi Noh, Muhammad Luqman Haider, Jeong Bong Lee, Rhonda D. Prisby

**Affiliations:** 1 Department of Kinesiology, The University of Texas at Arlington, Arlington, TX 76019, USA; nxs9024@mavs.uta.edu (N.S.H.);; 2 Department of Electrical Engineering and Computer Engineering, The University of Texas at Dallas, Richardson, TX 75080, USA; muhammadluqman.haider@utdallas.edu; 3 Department of Electrical and Computer Engineering, Baylor University, Waco, TX 76798, USA; jb_lee@baylor.edu

**Keywords:** young, old, intramedullary pressure, interstitial fluid flow, bone formation, micropump, wireless pressure sensor, microct, vascular function

## Abstract

Mimicking vascular function in bone may serve to stimulate osteogenesis. We developed a micropump and wireless pressure sensing system to fluctuate and record intramedullary pressure (IMP) in the femoral diaphysis of young (6 months) and old (24 months) male Fischer-344 rats. In vivo IMP was determined (*n* = 5 per group). Additionally, two bone defects were created in the right femora (EXP) of young (*n* = 14) and old (*n* = 11) rats and catheterized to the micropump and wireless pressure sensor. Intramedullary pressure was fluctuated for 10 min. The left femur served as the control (CTL). Seven days post-surgery, femora were scanned by μCT (15 µm) to assess trabecular bone microarchitecture, cortical thickness and new bone volume at the endosteal and periosteal surfaces. Data were analyzed with SPSS software, and significance was *p* ≤ 0.05. In vivo IMP was lower (*p* < 0.05) in the old (~16 mmHg) vs. young (~36 mmHg) rats. Trabecular thickness in the proximal metaphysis was higher (*p* < 0.05) in the old rats, and the cortical shell was thicker (*p* < 0.05) and new bone volume greater (*p* < 0.05) in the young rats. Importantly, new bone volume was greater (*p* < 0.05) in young EXP (14 ± 7 mm^3^) vs. young CTL (7 ± 7 mm^3^) femora. A 10-min bout of IMP fluctuation stimulated osteogenesis in young rats.

## 1. Introduction

Wolff’s Law characterizes how the structural arrangement of bone is reflective of its mechanical environment [1]. Mechanical loading [2,3] and reduced mechanical loading [4] that are chronically implemented, augments and diminishes bone mass, respectively. Thus, bone remodeling is regulated by mechanical loading [5] and is tightly coordinated by the activities of osteocytes, osteoblasts, and osteoclasts, each responding to biochemical and mechanical signals to preserve skeletal integrity [6,7,8]. Housed in lacunae and embedded in the mineralized matrix, osteocytes (i.e., the most abundant bone cell) are highly mechanosensitive and resistant to apoptosis, and undergo apoptosis with and without mechanical loading, respectively [9,10,11]. However, experimental evidence demonstrates that bone can adapt with minimal or no strain on the matrix. The magnitude of microstrain (με) experienced by bone determines the response. In other words, mechanotransduction requires 1000 to 5000 με [12]; however, to stimulate osteogenesis at the cellular level, ~10,000 με are required [13]. Several investigators have demonstrated that osteocytes are more responsive to fluid shear stress than to mechanical stretching at macroscopic strain levels [14,15,16,17] and the mechanical stimulus is perceived when mechanosensors in osteocytes are exposed to fluid flow [18]. Thus, mechanical stimuli are not only characterized as deformation of the peri-lacunar bone matrix but also by shear stress resulting from fluid flow [18]. Given the highly porous nature of the skeleton [19], interstitial fluid flows through the marrow and lacunar–canalicular network [20] with ease, subjecting bone (i.e., osteocytes, osteoblasts and osteoclast) and marrow cells to pressure changes and interstitial fluid flow-induced shear stress along their surface membranes. Thus, bone deformation may propagate the mechanical signal, but it is not the direct stimulus for adaptation.

Bone anabolism and/or catabolism [21] is the product of the release of factors [22,23,24] that augment or diminish bone cellular activity [25,26,27]. For example, following subjugation to fluid shear stress, bone-derived cells and organ cultures [22,28] released nitric oxide (NO) and prostaglandins (e.g., PGE_2_). Nitric oxide stimulates osteoblast mitosis [25,26] and inhibits osteoclast activity [27,29,30,31] and PGE_2_ augments bone formation and reduces immobilization-induced bone loss [32]. In fact, augmenting flow-induced shear stress led to the release of NO from osteoblasts, which was not elicited with mechanical strain [33]. Furthermore, subjecting unloaded turkey ulnae to oscillatory IMP loading in the absence of bone matrix strain resulted in bone accrual in the diaphysis [34]. Therefore, changes in intramedullary pressure can be utilized to initiate bone formation as well.

Skeletal fragility represents a major and growing clinical challenge, particularly in the context of an aging global population. In the United States alone, more than 10 million individuals were diagnosed with osteoporosis in 2010, while an additional 43 million exhibited low bone mass, placing them at elevated risk of fracture and reduced skeletal repair [35,36]. Aging is associated with decreased trabecular connectivity, thinning of the cortical shell, and diminished regenerative capacity; all of which collectively reduce bone strength and compromise the bone’s ability to heal after injury [37]. Given the highly fluidic nature of the skeleton and its reliance on IMP fluctuation and interstitial fluid flow for bone remodeling, participation of the vascular system in bone health and disease becomes paramount. Marrow capillaries provide interstitial fluid to the skeleton [38] and the bone nutrient arteries regulate blood flow and IMP [39]. Furthermore, the differential between IMP and systemic blood pressure allows for the exchange of fluid from the capillary into the interstitial space and lacunar–canalicular network [21]. Age-related vascular decline has been well documented in the form of reduced vasomotor function [40] and rarefaction of bone blood vessels [41,42], arterio- and atherosclerosis [43,44,45], and bone marrow blood vessel ossification [42]. These vascular pathologies reduce skeletal blood flow [3,40,46,47] and fluid volume [48,49] within the skeleton, and presumably diminish shear stress-induced release of bone remodeling factors. To test the hypothesis that controlled IMP modulation can serve as a non-pharmacological strategy to stimulate bone formation, especially in the context of advancing age, we developed and fabricated a micropump and wireless pressure sensing system [50,51,52]. Following acute IMP fluctuation, we assessed whether pressure-driven stimulation could induce bone anabolic responses and whether these responses differed between young and old rats.

## 2. Materials and Methods

### 2.1. Animals

All procedures were reviewed and approved by the Institutional Animal Care and Use Committee of the University of Texas at Arlington (IACUC Protocol A17.013) and adhered to the guidelines outlined in the Guide for the Care and Use of Laboratory Animals (NIH, eighth edition, 2011). Young (6 months old) and old (24 months old) male Fischer-344 rats were used for these series of investigations. Animals were housed in a temperature-controlled environment (23 ± 2 °C) under a 12:12 h light/dark cycle. Standard laboratory rat chow and tap water were available *ad libitum*.

### 2.2. Experiment 1

We previously reported on the development of a wireless pressure monitoring system with long-range communication [51]. The system consists of a wireless pressure sensor capable of detecting pressures ranging from 0 to 150 mmHg and a wireless transceiver interfaced to a computer program [51]. Three wireless pressure sensors (i.e., sensor 1, sensor 2 and sensor 3) were tested for validity and reliability against known pressures (cmH_2_O) of a hydrostatic column. Each wireless pressure sensor was tested independently via instrumentation to the hydrostatic column with a PE90 (1.27 mm or 0.050” diameter) catheter. The hydrostatic column was adjusted to three different heights corresponding to the following pressures chosen for validation: 9.5 cmH_2_O (~7 mmHg), 80 cmH_2_O (~59 mmHg), and 160 cmH_2_O (~118 mmHg), to encompass known IMP recordings from other investigations [53,54]. Following a 3-min equilibration period, pressure was recorded with the wireless pressure sensors. The last minute of the data was averaged and reported. The protocol was randomized among the wireless pressure sensors and performed two more times totaling three testing days. Given the known pressures of the hydrostatic column, we were able to determine the validity of each wireless pressure sensor (i.e., its ability to accurately read the pressures of the hydrostatic column). Furthermore, we assessed the reliability of each wireless pressure sensor by comparing the recorded data among the three testing days. Pressures are reported in mmHg.

### 2.3. Experiment 2

To assess in vivo IMP in the femoral diaphysis, the young (*n* = 5) and old (*n* = 5) rats were anesthetized (3% isoflurane to O_2_ balance for induction and 2.5% isoflurane to O_2_ balance to maintain the surgical plane). A bone defect was created in the femoral diaphysis (~3 mm from the knee joint) with a 20-gauge (0.76 mm or 0.030” diameter) needle. A polyethylene 50 (PE50; 0.965 mm or 0.038” diameter) catheter filled with 0.9% heparinized saline was advanced minimally into the bone defect and secured with surgical glue to prevent movement of the catheter and leaking of the marrow contents. The PE50 catheter was interfaced with the PE90 catheter of the wireless pressure sensor. In total, three wireless pressure sensors (i.e., sensor 1, sensor 2 and sensor 3) were tested in the young and old rats and the testing protocol was randomized. For each wireless pressure sensor, 5 min of IMP data was recorded, and the last 3 min of data were averaged and reported. Pressures are reported in mmHg.

### 2.4. Experiment 3

**Figure 1:**
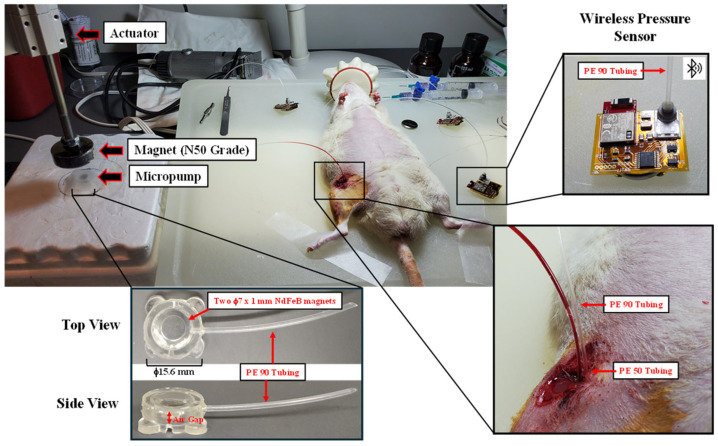
Schematic of the surgical and experiment setup illustrating two catheterized bone defects in the rat femur. The micropump was instrumented to a catheter inserted into the femoral canal and fluctuation of IMP was achieved via an external magnetic actuator. The wireless pressure sensor, instrumented to the second cathether, simultaneously enabled real-time monitoring of the changes in IMP.

To determine whether the micropump-induced IMP fluctuations sufficient to elicit bone formation, the young (*n* = 14) and old (*n* = 11) rats were anesthetized with isoflurane (3% to O_2_ balance for induction and 2.5% isoflurane to O_2_ balance to maintain the surgical plane). The skin overlaying the medial aspect of the distal femur was cut to expose the underlying musculature. Blunt dissection of the overlaying musculature was performed until the femoral diaphysis at the distal end of the bone was observed. A 20G needle (0.76 mm or 0.030”) was used to create two bone defects in the femoral diaphysis ~3 mm and ~8 mm above the knee joint. Two catheters (i.e., PE50) filled with 0.9% heparinized saline solution were inserted into the bone defects, advanced minimally into the intramedullary canal, and secured in place with surgical glue. Subsequently, the PE50 catheter was interfaced with PE90 catheters of the micropump and wireless pressure sensor. Care was taken to ensure that all connections were leak-free to maintain a pressurized condition. Prior to activating the micropump, baseline IMP was recorded for one minute. Once the pump was activated, IMP fluctuations were induced via a reciprocating external magnet as described previously [51]. The micropump was activated for 10 min and IMP was recorded with the wireless pressure sensor. Phosphate-buffered saline solution was used to keep the surgical area moist during the protocol. Following deactivation of the micropump, IMP was recorded for 1 min during recovery. The catheters were removed, the bone defects were sealed with surgical glue, and the skin was closed with a 4-0 suture. Left femora received the same surgical procedure as the right, absent catheterization and IMP fluctuation. Thus, the left femora served as the contralateral control. Figure 1 depicts the experimental setup. Following the surgical procedure on the left femora, the rats were housed singly as they recovered from surgery. Following 7 days, the rats were sacrificed (i.e., removal of the myocardium) while under anesthesia (3% to O_2_ balance). Right (EXP) and left (CTL) femora were dissected, cleaned of soft tissue, fixed in 10% formalin for 3 days, and stored in 70% EtOH.

### 2.5. Microcomputed Tomography (μCT)

Right and left femora were thawed at room temperature for an hour and subsequently scanned (15 μm) ex vivo and acquired (55 kVp) by μCT (Scanco Medical AG, Brüttisellen, Switzerland; MicroCT 45). To determine whether the induced fluctuations in IMP were effective throughout the femur or localized to the area of instrumentation (i.e., the distal femoral diaphysis), trabecular bone microarchitecture was evaluated in the secondary spongiosa of both the proximal and distal metaphyses. For each metaphyses, 76 slices were analyzed to quantify bone volume fraction (BV/TV, %), trabecular thickness (Tb.Th, μm), trabecular number (Tb.N, /mm), and trabecular separation (Tb.Sp, μm). Trabecular density (Tb.Density, mg HA/ccm) was also determined. Cortical bone was analyzed (30 slices) at the midshaft to assess cortical thickness (Ct.Th, μm) and cortical density (Ct.Density, mg HA/ccm). Cortical porosity (Ct.Porosity, %) was calculated as 1 − Ct.BV/TV × 100. Following μCT scanning of the whole femora, new bone formation was analyzed at the endosteal and periosteal surfaces, and the following parameters were reported: new bone volume (BV, mm^3^) and its bone mineral density (mg HA/ccm).

### 2.6. Statistical Analysis

An alpha level of *p* ≤ 0.05 was chosen *a priori*. For Experiment 1, One-Way ANOVA was used to determine reliability of the wireless pressure sensors among the testing days. For Experiment 2, One-Way ANOVAs were conducted to determine differences among IMP recordings of each wireless pressure sensor in the young and old groups independently and IMP between the young and old rats recorded by each wireless pressure sensor. For Experiment 3, One-Way ANOVA assessed differences in body mass and IMP fluctuations produced by the micropump between the young and old groups. In addition, trabecular bone microarchitecture and density, and cortical bone and new bone parameters were analyzed by Two-Way ANOVAs, with age (young vs. old) and treatment (CTL vs. IMP) as fixed factors. Pairwise comparisons (by use of the Least Significant Difference post hoc analyses) identified significant interactions in the data sets. Data are presented as Mean ± Standard Deviation (SD). The statistical analyses were conducted using SPSS software (Version 31, SPSS Inc, Armonk, NY, USA).

## 3. Results

### 3.1. Experiment 1

#### Reliability and Validity of the Wireless Pressure Sensors

Table 1 presents the mean pressure recordings from each wireless pressure sensor in comparison to the known pressures (i.e., ~7 mmHg, ~59 mmHg and ~118 mmHg) of the hydrostatic column. There were no significant differences in recorded pressure among testing day 1, testing day 2 and testing day 3. In addition, the pressures recorded by the wireless pressure sensors were similar to the known pressures of the hydrostatic column. Thus, each wireless pressure sensor was valid at reading the pressures of the hydrostatic column and each wireless pressure sensor reliably detected the pressures of the hydrostatic column on different testing days.

### 3.2. Experiment 2

#### Femoral IMP in Young and Old Rats

**Figure 2:**
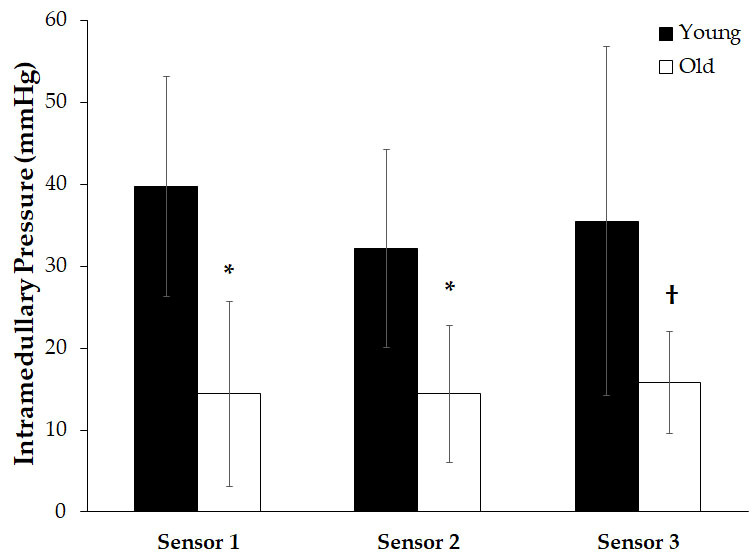
Intramedullary pressure (IMP) measured in the femoral diaphysis of young and old rats as recorded by three wireless pressure sensors. Values represent Mean ± S.D. * Denotes a significant (*p* < 0.05) difference between groups. † Denotes a tendency (*p* = 0.09) for difference between groups. *n* = 5 per group.

There were no differences in the IMP recordings among the wireless pressure sensors for the young and old rats. Table 2 presents the individual pressure data for each young and old rat and for each wireless pressure sensor. Intramedullary pressure was lower (*p* < 0.05) in the old vs. young rats as recorded by sensor 1 and sensor 2 (Figure 2). Furthermore, IMP tended (*p* = 0.09) to differ between the young and old rats as recorded by sensor 3.

### 3.3. Experiment 3

#### 3.3.1. Body Mass and IMP Fluctuations

Body mass was lower (*p* < 0.05) in the young (314 ± 23 g) vs. old (387 ± 18 g) rats and the mean IMP fluctuations generated by the micropump did not differ between the young (42 ± 26 mmHg) and old (46 ± 24 mmHg) groups.

#### 3.3.2. Trabecular Bone Microarchitecture and Density in the Proximal Metaphysis

No differences were observed in BV/TV, Tb.N, nor Tb.Sp; however, Tb.Th was higher (*p* < 0.05) in the old CTL and old EXP femora vs. the young CTL and young EXP femora (Table 3). In addition, Tb.Density was higher (*p* < 0.05) in old CTL and old EXP vs. young CTL and young EXP (Table 3).

#### 3.3.3. Trabecular Bone Microarchitecture and Density in the Distal Metaphysis

Trabecular bone microarchitectural properties (i.e., BV/TV, Tb.Th, Tb.N, and Tb.Sp) in the distal metaphysis did not differ within and between groups (Table 4). However, the trabeculae were denser (*p* < 0.05) in the old CTL and old EXP femora vs. the young CTL and young EXP femora (Table 4).

#### 3.3.4. Cortical Bone Parameters at the Midshaft

The cortical shell was thicker (*p* < 0.05) in the young vs. old rats; however, Ct.Density was higher in the old vs. young femora (Table 5). No differences in Ct.Porosity were observed.

#### 3.3.5. New Bone Formation at the Endosteal and Periosteal Surfaces

**Figure 3:**
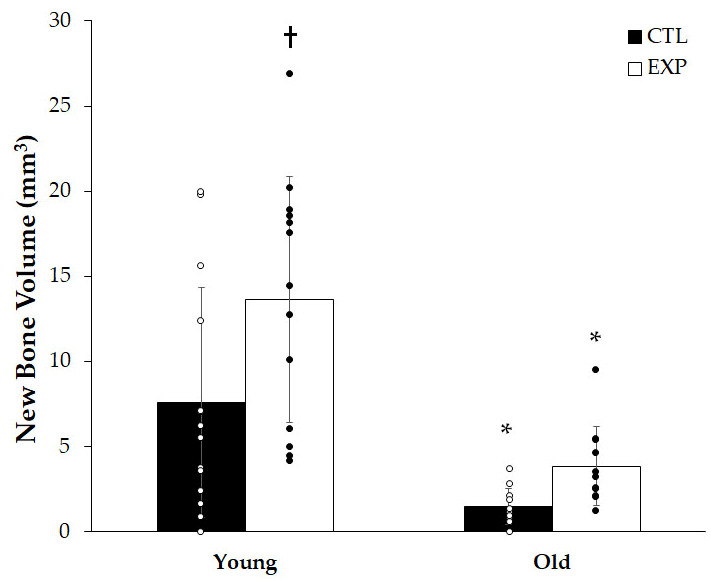
New bone volume (mm^3^) at the periosteal and endosteal surfaces of control (CTL) and experimental (EXP) femora in young (7 ± 7 mm^3^ vs. 14 ± 7 mm^3^, respectively) and old (1 ± 1 mm^3^ vs. 4 ± 2 mm^3^, respectively) rats. A two-fold increase was observed in young EXP vs. young CTL. Values represent Mean ± S.D. * Denotes a significant (*p* < 0.05) from the young groups. † Denotes a significant (*p* < 0.05) difference between young CTL and young EXP. young (*n* = 14); old (*n* = 11).

**Figure 4:**
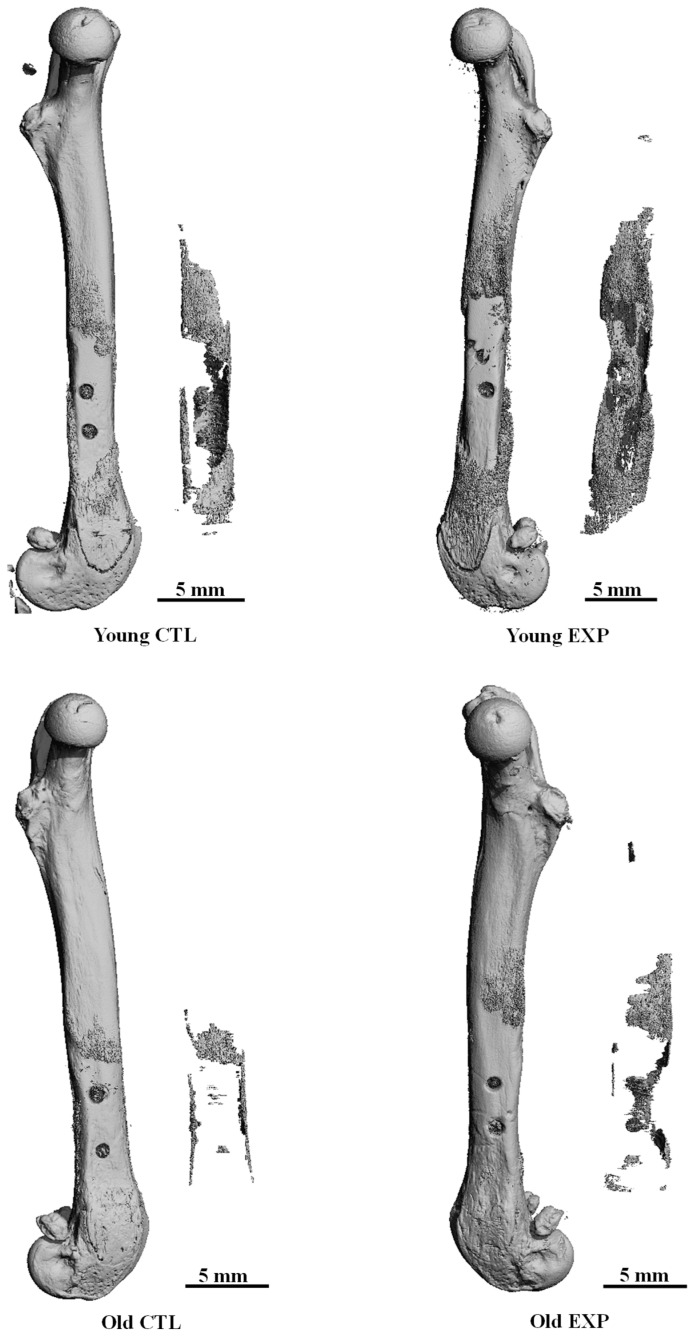
Representative 3D μCT reconstructions illustrating new bone volume at the periosteal and endosteal surfaces of young and old femora. Observable on the diaphyses are the two bone defects. The new bone volume can be observed in relation to the whole femora (**left**) and in isolation from the whole femora (**right**); i.e., the μCT analysis of only the new bone volume for each femur is visible. Note that the new bone volume is often a considerable distance from the bone defects. New bone volume was higher (*p* < 0.05; see Figure 3) in the young vs. old rats. In other words, the percentage differences between young CTL vs. old CTL and young EXP vs. old EXP are 150% and 115%, respectively. Additionally, the percentage difference in new bone volume between the young CTL vs young EXP is 67% (*p* < 0.05; see Figure 3).

New bone volume at the endosteal and periosteal surfaces was higher (*p* < 0.05) in the young CTL and young EXP femora vs. old CTL and old EXP femora (Figure 3); however, the new bone was less (*p* < 0.05) dense in the young vs. old CTL (627 ± 37 mg HA/ccm vs. 731 ± 104 mg HA/ccm, respectively) and young vs. old EXP (601 ± 42 mg HA/ccm vs. 712 ± 82 mg HA/ccm, respectively) femora. Finally, new bone volume was higher (*p* < 0.05) in the EXP (14 ± 7 mm^3^) vs. CTL (7 ± 7 mm^3^) femora in the young rats (Figure 3). Figure 4 depicts representative μCT images of new bone volume at the periosteal and endosteal surfaces of young and old femora.

## 4. Discussion

There are three major findings of these investigations: (1) the wireless pressure sensors were valid and reliable, (2) IMP was reduced in the old vs. young rats, and (3) a 10-min bout of IMP fluctuation enhanced periosteal and endosteal bone formation in young rats. Given the context of the experimental design, however, additional factors may have contributed to the osteogenic response and are discussed below. Among other age-related vascular pathologies, dysregulation of bone IMP and its presumed contribution to altered interstitial fluid flow may contribute to age-related bone loss. The literature is replete with novel strategies designed to augment bone formation. For example, cellular (i.e., sclerostin-LRP4 signaling and post-translational modifications [55,56]) and biomaterial-based (e.g., reinforced calcium phosphate and glass ionomer cement composites [57,58]) strategies have shown promising results. Despite these advances, most existing approaches focus on external loading, implanted biomaterials, or molecular modulation rather than reproducing the physiological role of the bone vascular system on IMP regulation. The findings herein support the use and further development of the fabricated micropump to induce IMP fluctuation as a non-pharmacological approach for bone anabolism.

Advancing age is associated with a plethora of physiological ailments, one among them being the development of osteoporosis [35,36] and enhanced fracture risk. Much attention in this regard has primarily focused on the cellular and molecular aspects of bone, while underappreciating the integrative nature of biological organisms. We posit that the vascular system plays an important role in bone biology [59]. In addition to the well-defined responsibilities (i.e., providing O_2_ and nutrients to and removing metabolic byproducts from tissues), the bone vascular network contributes to the efficient functioning of the skeleton. For example, bone blood vessels deliver systemic hormones and precursor cells for remodeling [60,61], participate in hematopoiesis [62], are vital components of basic multicellular units [63,64], are conduits for the egress and ingress of blood and immune cells to and from the marrow [65,66,67], and maintain the fluidic nature of the skeleton [7]. For example, bone and marrow capillaries provide interstitial fluid [38] and the bone nutrient arteries regulate skeletal blood flow and IMP [39]. Thus, when considering age-related bone pathology, alterations in the bone vascular network should be considered.

Several age-related decrements in the bone blood vessels have been reported, i.e., reduced endothelium-dependent vasodilation [40], vascular rarefaction [41], reduced angiogenic capacity [68], the centripetal direction of blood flow [69], arterio- and atherosclerosis [43,44,45], bone marrow blood vessel ossification [42], marrow ischemia [48], and reduced skeletal blood flow [40]. All of these would serve to reduce the fluid volume within the skeleton and presumably disrupt the mechanisms by which mechanical loading imparts its bone-regulating effects (i.e., via the alteration of IMP and interstitial fluid flow).

Skeletal IMP [70] and the pulse pressure of the marrow [71] vary according to the bone compartment and according to animal species. Intramedullary pressure recordings presented in the literature are highly variable, i.e., ranging from ~16 mmHg in mice [53] up to 60 mmHg in dogs [54]. The IMP induced by the micropump (i.e., 42 ± 26 mmHg and 46 ± 24 mmHg in the young and old rats, respectively) fall within this physiological range. Furthermore, IMP pulsation aids in promoting circulation throughout the skeleton [69]. Since fluid movement within the skeleton is vital for bone adaptation and osteocyte survival [21], factors that impair blood delivery to the skeleton (e.g., vascular pathologies) serve to reduced IMP and interstitial fluid flow. We report reduced IMP in the old vs. young rats, supportive of our hypothesis and contributing to the list of age-related vascular pathologies.

These investigations were undertaken to develop a non-pharmaceutical intervention capable of ameliorating or reversing age-related skeletal decline by mimicking the actions of the vascular system. These efforts led to the development and refinement of a prototype micropump and wireless pressure sensor system [50,51,52]. Herein, we report that the wireless pressure sensors were valid and reliable at measuring the changes in pressure. Moreover, we demonstrate that the fabricated micropump delivered controlled IMP fluctuations that are consistent with physiological ranges [53,54] and were within and higher than the mean IMP for the young and old rats, respectively. Despite augmenting IMP fluctuation in the old rats, bone anabolism was minimal. For example, IMP was ~16 mmHg in the old animals and the mean IMP induced by the micropump was 46 mmHg, i.e., a mean increase of 2.9-fold. While bone formation was observed at the endosteal and periosteal surfaces, bone volume was similar between the old EXP and old CTL femora. In contrast, IMP was ~36 mmHg in the young animals and the mean IMP induced by the micropump was 42 mmHg, i.e., a 6-mmHg difference. Despite this small difference, bone volume at the endosteal and periosteal surfaces was augmented in the young EXP vs. CTL femora. Other investigations demonstrated similar findings. For example, dynamic hydraulic stimulation (i.e., the elevation of IMP absent large changes in με), augmented endosteal bone formation [72], cyclic hydrostatic pressure induced osteogenic differentiation of human bone-marrow–derived stem cells [73], and microfluidic enhancement of IMP elevated interstitial fluid flow and inhibited bone loss in hindlimb-suspended mice [53]. While not measured in this investigation, it is reasonable to assume that the IMP fluctuations elicited changes in interstitial fluid flow within the femora.

The endosteal and periosteal surfaces of the femora demonstrated osteogenic responsiveness in both the young and old rats, which was not limited to areas adjacent to the sub-critical bone defects and often extended to the proximal end (Figure 4). This bone anabolic adaptation was observed in both the EXP and CTL femora, exacerbated in the young vs. old rats, and in the young EXP vs. CTL. We speculate that the bone anabolic response can be attributed to one or more of the following mechanisms: (1) healing of the bone defects, (2) a compensatory response related to mechanical instability, (3) exposure of the periosteal surface to marrow cells, and/or (4) the IMP fluctuation induced by the micropump. While the definition of a sub-critical sized defect varies according to the anatomical location in mice, defects <0.80 mm wide heal without intervention [74,75,76]. Even though they heal without intervention, the bone biomechanics (e.g., resistance to bending, twisting and compression) can be temporarily altered, interfering with the even distribution of load across the bone. Thus, sub-critical bone defects can reduce mechanical stability in a transient fashion. The bone defects created in this study penetrated the full thickness of the cortex, were 0.76 mm in diameter, and were ~5 mm away from one another. Thus, the defects in our rat model were sub-critical in nature and some of the new bone volume undoubtedly relates to the healing process. Furthermore, to compensate for the mechanical instability resulting from the two bone defects, bone may have formed at locations experiencing augmented με. This may account for the new bone volume observed at locations distant from the bone defects. The loads experienced by rat femora equals to ~½ of the body mass [77]. Interestingly, the old rats had higher body masses in comparison to the young, potentially augmenting the mechanical instability resulting from the bone defects. Yet, the bone anabolic response was weaker in comparison. The contribution of healing and mechanical instability to the new bone volume can be further contextualized using the CTL data. Because the CTL femora also received two bone defects but no IMP fluctuation, the new bone volume measured in the CTL limbs (young: 7 ± 7 mm^3^ and old: 1 ± 1 mm^3^) reflects a combination of potential factors (e.g., systemic immune influences, defect healing and augmented με) that are independent from the stimuli of the micropump.

Furthermore, the bone anabolic response may have been influenced by the presence of marrow cells at the periosteal surface. The creation of two bone defects during the surgical procedure allowed for this possibility. For example, a recently discovered skeletal stem cell may explain the weaker anabolic response in the old rats. Skeletal stem cells reside in the marrow and are important regulators of bone formation [78]. During creation of the bone defects, skeletal stem cells may have escaped the marrow and initiate bone formation at the periosteal surface. Young marrow is replete with Fgfr3^+^ endosteal skeletal stem cells, which serve as a hearty source of osteoblasts and participate in normal and abnormal bone formation [78]. These cells are depleted in aged marrow [78] and may partly explain the reduced anabolic response.

The final possibility is that the intervention was successful at augmenting bone mass. The enhanced bone volume at the endosteal and periosteal surfaces of the young EXP vs. young CTL indicates that a single 10-min IMP session with the micropump was sufficient to trigger an anabolic response in young bone. The augmented new bone volume observed in the young EXP femora (14 ± 7 mm^3^), i.e., a twofold increase vs. young CTL, arguably represents the micropump-driven IMP fluctuation superimposed upon and surpassing the other presumed contributing factors. These data are consistent with a previous report of bone accrual in the diaphysis of unloaded turkey ulnae in response to oscillation of IMP [34]. Additionally, we theorized that the IMP fluctuations may have generated interstitial fluid flow through the marrow and the lacunar–canalicular network, exposing osteocytes, osteoblast and osteoclasts to enhanced pressure and fluid shear stress.

In the old rats, where EXP and CTL new bone volume did not differ, one could argue that the contribution from IMP fluctuation was negligible. This pattern indicates that the two bone defects were sufficient to initiate an osteogenic response at both the periosteal and endosteal surfaces consistent with the influences of the surgical intervention, localized healing, and/or augmented με as discussed above. The lack of a significant anabolic difference between the old EXP and old CTL femora may stem from several non-mutually exclusive mechanisms. First, the micropump-induced IMP fluctuations were not of sufficient strength to elicit an effective bone response in the old rats. Second, the aged bone cells may be dysfunctional, leading to an inequivalent biological response to the stimuli, as reported in previous studies [79]. Third, the 7-day recovery period may not have been of sufficient duration to capture a delayed or protracted osteogenic response in old bone, given the well-documented decline in cellular activity and bone remodeling rates with aging. The proposed mechanisms, while plausible, were not directly investigated in the present study. Therefore, the underlying cellular and molecular processes remain to be elucidated. Regardless, the same IMP stimulus that initiated an anabolic response in young bone failed to surpass the activation threshold needed for old bone. In addition, new bone volume was lower in both old femora vs. the young femora, underscoring the intrinsic age-related decline in regenerative potential. The enhanced intrinsic bone formation observed in the young rats is consistent with evidence that youthful bone maintains higher mesenchymal stem-cell proliferation and differentiation capacity, a more favorable IGF-1 hormonal profile, and greater angiogenic support [80]. In contrast, aging is associated with oxidative stress, cellular senescence, and impaired vascular signaling, which collectively diminishes repair and remodeling capacity.

Given the acute (i.e., 10 min of IMP fluctuation) nature and short time frame (i.e., 7 days) of the intervention, we did not anticipate changes in trabecular bone microarchitecture (i.e., BV/TV, Tb.Th, Tb.N and Tb.Sp) at the proximal and distal metaphyses or cortical bone parameters (i.e., Ct.Th and Ct.Porosity) at the midshaft as measured by μCT analysis. However, the biological significance of new bone formation at the periosteal and endosteal surfaces, particularly in the young EXP femora which surpassed that in young CLT, should not be overlooked. We have previously discussed other potential contributing factors to this rapid osteogenesis. However, the exacerbated new bone volume in the young EXP vs CTL femora provide evidence of the effectiveness of this intervention. Given the 7-day protocol, which is insufficient in duration to elicit volumetric changes in trabecular bone microarchitecture and cortical bone structure, osteogenesis related to the IMP fluctuations occurred. These data are encouraging and support the notion that this non-pharmacological intervention may be efficacious. Modifications to the experimental protocol (i.e., extension of the recovery period, increasing the duration of the IMP fluctuations and/or repeated delivery of the IMP fluctuations over weeks to months, etc.) may provoke the desired alterations in trabecular bone microarchitecture and/or cortical bone structure in both young and old femora. Furthermore, our findings are consistent with Robling et al. (2002) who similarly reported that short-term or single mechanical loading bouts stimulate early bone matrix formation but do not produce measurable gains in mineral content or microarchitecture unless the loading is sustained or repeated over time [12]. Similarly, the increase in periosteal and endosteal new bone volume likely represents an early anabolic response preceding any structural changes in trabecular and cortical bone. Thus, the absence of significant changes in trabecular bone microarchitecture and cortical bone structure does not diminish the biological significance observed at the periosteal and endosteal surfaces, instead it highlights the importance of the vascular system in bone biology. In other words, by simply mimicking the role of bone blood vessels in the regulation of IMP, osteogenesis can be achieved via non-pharmacological means. Even though BV/TV, Tb.Th and Tb.Sp did not differ between the young and old animals, trabeculae were thicker in the proximal metaphysis of the old rats. Additionally, the midshaft cortical shell was thicker in the young rats. In contrast, trabecular bone density in the proximal and distal metaphyses and cortical density at the midshaft were higher in the old vs. young femora. Higher trabecular and cortical bone densities in the Fisher-344 rat model are consistent with previous reports in the literature [81] and represent age-related changes.

A key limitation of this study is that the acute intervention (i.e., a single 10 min bout) was not sufficient to elicit adaptations in trabecular and cortical bone. This protocol was a methodological choice, informed by the technical constraints of our novel platform. At the time of these studies, the micropump design was still in its infancy stage and these protocols served as proof-of-concept studies. With further refinement of the device, longer duration protocols are forthcoming. An additional limitation is that the left femur served as the contralateral CTL. Thus, bone defects were created but the left femora did not receive catheterization or IMP fluctuation. The use of the left femur as the contralateral CTL minimized inter-animal variability (e.g., body mass, genetic background, baseline bone characteristics, etc.). However, we acknowledge that the surgical intervention may have elicited systemic responses capable of influencing both limbs. Therefore, although this within-animal design reduced biological variability, systemic effects cannot be completely excluded. Furthermore, since the CTL femur did not undergo catheterization, the potential influence of catheterization on marrow perturbance and subsequent bone remodeling cannot be entirely separated from the bone remodeling resulting from IMP fluctuation, even though the catheters were advanced to a minimal degree. Future studies incorporating independent sham-operated groups with catheterization, but absence of pump activation, may better isolate the specific contribution of IMP fluctuation on bone formation. While these studies relied upon μCT as the endpoint analysis, bone histomorphometry is currently underway to assess bone cellular events that may have occurred within the 7-day recovery period. Furthermore, although IMP fluctuation is proposed to stimulate bone formation by enhancing interstitial fluid flow, fluid flow was not measured directly in the present study and therefore this mechanism remains inferential. Finally, the scope of this investigation was limited to male rats. Given the well-documented sex differences in bone metabolism and remodeling rates, the findings presented herein may not be indicative of both sexes. Future studies will therefore include both male and female cohorts to determine whether sex-dependent differences exist in the osteogenic response to controlled IMP fluctuations, particularly in the context of aging. In addition, longer-term and repeated IMP fluctuation protocols will be implemented in both young and old, and male and female animals to determine whether sustained stimulation can overcome the reduced sensitivity observed in aged bone and ultimately promote trabecular and/or cortical bone adaptation. Collectively, these next steps will allow separation of injury-driven repair responses from pressure-induced osteogenesis and will strengthen the translational relevance of this platform for age-related bone loss, osteoporosis-related fragility, and perhaps fracture repair.

## 5. Conclusions

In summary, the wireless pressure sensors were valid and reliable, IMP was reduced in old rats, and a single 10-min session of IMP fluctuation augmented new bone formation on the periosteal and endosteal surfaces of femora in the young but not the old rats. Thus, experimental protocols designed to mimic the activities of the bone vascular system (i.e., the regulation of IMP) may serve as a therapeutic means to augment bone mass. It must be noted, however, that within the context of the present experimental design, the new bone formation cannot be attributed solely to the IMP fluctuations. These findings should be interpreted within the context of a surgical bone defect model, in which IMP fluctuation occurred alongside other potential biological factors (i.e., bone healing, systemic inflammatory responses, etc.) and transient mechanical instability. The lack of adaptation in the old animals may reflect reduced mechanosensitivity, which warrants further investigation. These results underscore the importance of considering the role of vascular function in bone remodeling in youth and old age, particularly since the biological context of aging often shifts skeletal and vascular dynamics towards less favorable outcomes. Future investigations will focus on longer durations of IMP modulation to stimulate bone formation in the old animals and at the trabecular and cortical compartments in both sexes and age groups.

## Figures and Tables

**Table 1:** Validity and reliability of the wireless pressure sensors.

	Testing Day 1	Testing Day 2	Testing Day 3	Hydrostatic Column Pressure (mmHg)
Sensor 1 (mmHg)	9	9	8	~7
	60	59	58	~59
	116	116	116	~118
Sensor 2 (mmHg)	7	7	10	~7
	56	57	59	~59
	113	113	114	~118
Sensor 3 (mmHg)	8	10	10	~7
	56	60	57	~59
	112	117	111	~118

Values represent the average pressure (mmHg) of the last minute of a 3 min recording. There were no differences in pressure among testing days as recorded by the wireless pressure sensors. Furthermore, pressures recorded by the wireless pressure sensors were similar to the known pressure of the hydrostatic column.

**Table 2:** Femoral intramedullary pressure recordings with the wireless pressure sensors.

	Sensor 1 (mmHg)	Sensor 2 (mmHg)	Sensor 3 (mmHg)
Young 1	35 ± 1	35 ± 3	30 ± 3
Young 2	58 ± 3	44 ± 3	67 ± 3
Young 3	*	13 ± 2	*
Young 4	26 ± 2	29 ± 4	22 ± 1
Young 5	40 ± 2	40 ± 1	23 ± 3
	Sensor 1 (mmHg)	Sensor 2 (mmHg)	Sensor 3 (mmHg)
Old 1	22 ± 1	22 ± 1	15 ± 2
Old 2	23 ± 2	16 ± 1	12 ± 1
Old 3	10 ± 1	9 ± 1	10 ± 1
Old 4	35 ± 3	22 ± 2	26 ± 1
Old 5	7 ± 1	3 ± 1	16 ± 1

Values are Means ± S.D. * indicates a loss of detection during the test.

**Table 3:** Trabecular bone microarchitecture and density in the proximal femoral metaphysis.

	YOUNG (*n* = 14)		OLD (*n* = 11)	
	CTL (*n* = 14)	EXP (*n* = 13)	CTL (*n* = 11)	EXP (*n* = 11)
BV/TV (%)	28 ± 1	28 ± 1	29 ± 1	28 ± 1
Tb.Th (µm)	116 ± 2	115 ± 2	124 ± 2 *	123 ± 2 *
Tb.N (1/mm)	1.99 ± 0.05	1.96 ± 0.05	1.95 ± 0.06	1.99 ± 0.06
Tb.Sp (µm)	515 ± 17	530 ± 17	514 ± 21	532 ± 20
Tb.Density (mg HA/ccm)	1039 ± 2	1036 ± 2	1082 ± 3 *	1084 ± 3 *

Values represent Mean ± SD. * Denotes a significant (*p* < 0.05) difference vs. young CTL & young EXP.

**Table 4:** Trabecular bone microarchitecture and density in the distal femoral metaphysis.

	YOUNG (*n* = 14)		OLD (*n* = 11)	
	CTL (*n* = 14)	EXP (*n* = 13)	CTL (*n* = 11)	EXP (*n* = 11)
BV/TV (%)	18 ± 1	15 ± 1	16 ± 1	16 ± 1
Tb.Th (µm)	89± 3	81 ± 3	88 ± 4	88 ± 4
Tb.N (1/mm)	2.53 ± 0.09	2.44 ± 0.10	2.28 ± 0.11	2.35 ± 0.11
Tb.Sp (µm)	401 ± 17	412 ± 18	432 ± 20	421 ± 20
Tb.Density (mg HA/ccm)	1030 ± 4	1038 ± 4	1084 ± 4 *	1081 ± 4 *

Values represent Mean ± S.D. * Denotes a significant (*p* < 0.05) difference vs. young CTL & young EXP.

**Table 5:** Cortical bone parameters at the midshaft.

	YOUNG (*n* = 14)		OLD (*n* = 11)	
	CTL (*n* = 14)	EXP (*n* = 13)	CTL (*n* = 11)	EXP (*n* = 11)
Ct.Th (μm)	550 ± 20	554 ± 20	452 ± 20 *	452 ± 25 *
Ct.Porosity (%)	27 ± 25	28 ± 26	14 ± 14	18 ± 19
Ct.Density (mg HA/ccm)	1198 ± 17	1193 ± 16	1269 ± 16 *	1265 ± 17 *

Values represent Mean ± S.D. * Denotes a significant (*p* < 0.05) difference from the respective young groups.

## Data Availability

The data is available upon valid request to the corresponding author.

## Abbreviations

The following abbreviations are used in this manuscript:

IMP	Intramedullary Pressure
kVp	Kilovoltage Peak
BV/TV	Bone Volume Fraction
Tb.Th	Trabecular Thickness
Tb.N	Trabecular Number
Tb.Sp	Trabecular Separation
Tb.Density	Trabecular Density
Ct.Th	Cortical Thickness
Ct.Density	Cortical Density
Ct.Porosity	Cortical Porosity
BV	Bone Volume
CTL	Control
EXP	Experimental
g	Grams
mmHg	Millimeters of Mercury
μm	Microns
mgHA/ccm	Milligrams of Hydroxyapatite per Cubic Centimeter
με	Microstrain
NO	Nitric Oxide
PGE_2_	Prostaglandin E_2_
IACUC	Institutional Animal Care and Use Committee
°C	Degrees Celsius
h	Hour
cmH_2_O	Centimeters of Water
mm	Millimeter
”	Inches
~	Approximately
%	Percent
O_2_	Oxygen
PE	Polyethylene
G	Gauge
4-0	4 Ought
S.D.	Standard Deviation
